# The Structural Basis of Long-Term Potentiation in Hippocampal Synapses, Revealed by Electron Microscopy Imaging of Lanthanum-Induced Synaptic Vesicle Recycling

**DOI:** 10.3389/fncel.2022.920360

**Published:** 2022-08-01

**Authors:** John E. Heuser

**Affiliations:** Department of Cell Biology and Physiology, Washington University School of Medicine, St. Louis, MO, United States

**Keywords:** hippocampal synapses, synaptic vesicle, endocytosis, lanthanum, long-term potentiation, postsynaptic densities (PSDs)

## Abstract

Hippocampal neurons in dissociated cell cultures were exposed to the trivalent cation lanthanum for short periods (15–30 min) and prepared for electron microscopy (EM), to evaluate the stimulatory effects of this cation on synaptic ultrastructure. Not only were characteristic ultrastructural changes of exaggerated synaptic vesicle turnover seen within the presynapses of these cultures—including synaptic vesicle depletion and proliferation of vesicle-recycling structures—but the overall architecture of a large proportion of the synapses in the cultures was dramatically altered, due to large postsynaptic “bulges” or herniations into the presynapses. Moreover, in most cases, these postsynaptic herniations or protrusions produced by lanthanum were seen by EM to distort or break or “perforate” the so-called postsynaptic densities (PSDs) that harbor receptors and recognition molecules essential for synaptic function. These dramatic EM observations lead us to postulate that such PSD breakages or “perforations” could very possibly create essential substrates or “tags” for synaptic growth, simply by creating fragmented free edges around the PSDs, into which new receptors and recognition molecules could be recruited more easily, and thus, they could represent the physical substrate for the important synaptic growth process known as “long-term potentiation” (LTP). All of this was created simply in hippocampal dissociated cell cultures, and simply by pushing synaptic vesicle recycling way beyond its normal limits with the trivalent cation lanthanum, but we argued in this report that such fundamental changes in synaptic architecture—given that they can occur at all—could also occur at the extremes of normal neuronal activity, which are presumed to lead to learning and memory.

## Introduction

Of all the important structural features of the synapse that have been viewed in the electron microscope over the decades, a feature that has been the most correlated with long-term potentiation (LTP), especially in the context of electron microscopy (EM) preparations generated from hippocampal tissues that were experimentally manipulated into LTP conditions, would have to be the *perforated postsynaptic density* (or *perforated PSD*).

It was the research work of one of the leading electron microscopists of the hippocampus, Yuri Geinesman, to establish this correlation (Geinisman et al., 1986, 1987a,b, 1991, 1992a,b, 1993, 1994, 1996; Geinisman, 1993, 2000). Another top electron microscopist of the hippocampus, Kirsten Harris, reported *perforated PSD’s* almost as often, but remained more agnostic about their role in hippocampal LTP (Harris and Stevens, 1989; Chicurel and Harris, 1992; Spacek and Harris, 1997; Sorra et al., 1998; Fiala et al., 2002; Harris et al., 2003; Spacek and Harris, 2004; Bourne and Harris, 2011; Bailey et al., 2015; Watson et al., 2016). Likewise, two other top EM labs that published a lot on the hippocampus, Michael Stewart’s in Milton Keynes, England (Dhanrajan et al., 2004; Popov et al., 2004; Stewart et al., 2005a,b; Medvedev et al., 2010), and Dominique Muller’s in Geneva, Switzerland (Stoppini et al., 1991; Toni et al., 1999, 2001; Luscher et al., 2000), reported finding *perforated PSDs* in a lot of publications and also made outstanding contributions toward understanding how these might be involved in LTP or “synaptic learning” (Stoppini et al., 1991; Toni et al., 1999, 2001; Luscher et al., 2000; Dhanrajan et al., 2004; Popov et al., 2004; Stewart et al., 2005a,b; Medvedev et al., 2010). All this history culminated with the later, outstandingly beautiful cryo-EM work on this same topic, which came from Michael Frotscher’s lab in Freiberg, Germany (Zhao et al., 2012a,b,c). Along the way, and right up to the present day, many other EM labs have weighed in with valuable correlative data and their own ideas on how LTP might come about (Tarrant and Routtenberg, 1977; Calverley and Jones, 1987a,b; Petit et al., 1989; Markus et al., 1994; Neuhoff et al., 1999; Kennedy, 2016, 2018; Sun et al., 2021), and numerous reviews of perforated synapses have been published (although without any serious attempt to correlate them with learning or memory) (Petralia et al., 2015, 2016, 2017, 2018, 2021).

At hippocampal synapses, the postsynaptic densities or PSDs are ordinarily disk-shaped entities that are generally located directly across from presynaptic neurotransmitter release sites (Gray, 1959, 1976; Blomberg et al., 1977; Cohen et al., 1977; Cohen and Siekevitz, 1978; Carlin et al., 1980; Dosemeci et al., 2001; Tao-Cheng et al., 2009; High et al., 2015). They generally appear almost *continuous* in thickness and density across the breadth of the disk, and even though recent close inspections have begun to suggest that each PSD disk may have a sub-structure, and may be composed of sub-modules or “nanomodules” (MacGillavry et al., 2013; Broadhead et al., 2016; Zeng et al., 2016; Biederer et al., 2017; Chen et al., 2018, 2020; Crosby et al., 2019; Trotter et al., 2019; Obashi et al., 2021; Ramsey et al., 2021; Wegner et al., 2022), these PSD components pack closely enough together to create the general impression in the EM of a continuous plaque or a disk.

“Perforated” PSDs, in contrast, are variable in outline. They look more like irregular and discontinuous patches in the EM, but they are presumed to be composed of the same components that are found in the more normal, continuous PSD disks. Reviewing the aforementioned references, one can find a wide range of thoughts about them, everything from conclusions that such “perforated” PSDs are in the process of division of one plaque into two (ultimately to form two different postsynaptic spines, in some of the bolder claims), to conclusions that they are simply a by-product of synaptic activity. But overall, “perforated” PSDs have generally been interpreted as synapses-in-augmentation, as would be expected if they were the structural correlates of the synaptic enhancement that is presumed by everyone to be the fundamental basis of LTP (Bliss and Gardner-Medwin, 1973; Bliss and Lomo, 1973; Carlin and Siekevitz, 1983; Lømo, 2003, 2018; Bailey et al., 2015).

In this report, we presented serendipitous observations that could help to explain exactly how “bursts” of presynaptic activity could cause perforated PSDs to form, in the first place. Specifically, we argued that these bursts of presynaptic activity could create delays in the process of synaptic vesicle recycling, which could make the presynapse expand in surface area, and do in such a manner that it could actually strain and break the normal plaque-like PSD into a “perforated” PSD.

Our EM images were obtained from primary (dissociated cell) hippocampal cultures prepared by classical techniques (Skrede and Westgaard, 1971; Schwartzkroin and Wester, 1975; Ransom et al., 1977; Mayer et al., 1989; Lu et al., 1998; Scott et al., 2001; Blanpied et al., 2002). These were chemically stimulated *via* direct application of low doses (0.1 mM) of the trivalent cation, lanthanum (La + + +). This magical trivalent cation has been used to stimulate spontaneous neurosecretion in many different preparations over the past half century (Miledi, 1971; Curtis et al., 1986; Coniglio et al., 1993; Powis et al., 1994; Powis and Clark, 1996; Lerner et al., 2006; Chung et al., 2008; Wasser and Kavalali, 2009; Ramirez and Kavalali, 2011), but still, no one knows exactly *how or why* it does so, especially because it is generally considered to be a “super calcium” that actually *blocks* most calcium channels, and thus blocks most calcium-evoked neurosecretory phenomena, it does not *stimulate* them (dos Remedios, 1981; Lansman, 1990; Reichling and MacDermott, 1991; Meir et al., 1998).

When we first faced this conundrum 50 years ago (the conundrum of why lanthanum blocks calcium-induced neurosecretion but hugely stimulates *spontaneous* transmitter release, in the form of huge bursts of miniature endplate potentials or “m.e.p.p.’s” at the NMJ), we simply could not explain it, but nevertheless we “used” the phenomenon to deplete synaptic vesicles from frog neuromuscular junctions, and thus to expose for the first time the resultant membranous transformations that turned out to represent synaptic vesicle recycling (Heuser and Miledi, 1971; Heuser, 1976, 1989a,b). Our scientific colleagues in those days quickly followed suit, and also used lanthanum to show that a dramatic expansion of the presynaptic membrane can accompany this spontaneous lanthanum-induced transmitter discharge, due to a slowdown of synaptic vesicle recycling and accumulation of vesicle membrane on the presynaptic surface (von Wedel et al., 1981; Jones et al., 1982; Segal et al., 1985; Robitaille and Tremblay, 1987; Torri-Tarelli et al., 1987, 1990; Valtorta et al., 1988). In fact, we intend to show here—by classical thin-section EM—that it is this presynaptic *expansion*, which is most dramatic in hippocampal cultures, and furthermore, that the unique distortion(s) which this expansion creates on the postsynaptic side of hippocampal synapses can greatly help to explain *how and why* their PSDs can become perforated during enhanced synaptic activity.

## Results

### Structural Evidence of Lanthanum’s Stimulatory Effects

A total of 57 separate experiments were performed with lanthanum on hippocampal cultures, adjusting the time of exposure (5–30 min), the dose of lanthanum (0.1–1 mM), the level of calcium counter ions (0–1 mM), and the method of fixation for EM (glutaraldehyde in cacodylate buffer vs. in Hepes buffer). Such classical dissociated cell hippocampal cultures invariably display a huge range of synaptic types, only vaguely reflecting the characteristic and stereotypical forms of synapses observed in the intact hippocampus or in hippocampal slices (Skrede and Westgaard, 1971; Schwartzkroin and Wester, 1975; Ransom et al., 1977; Mayer et al., 1989; Lu et al., 1998; Scott et al., 2001; Blanpied et al., 2002). Nevertheless, most of the synapses that manage to reform in such dissociated cell cultures fall into two major categories: bouton-like (e.g., onto dendritic protrusions that are generally described as primitive “spines”), and plague-like (e.g., directly onto the shafts of the primitive, disoriented dendrites in such cultures). The former types of synapses typically have single PSD plagues that are generally considered to be “excitatory.” These are the so-called “*asymmetric*” synapses, due to their relatively thick PSDs. The latter typically display 2 or 3 adjacent plaques in the dendritic shaft and are generally considered “inhibitory.” These are the so-called “*symmetric*” synapses, due to their relatively thin and often barely perceptible PSDs (Gray, 1959, 1976; Blomberg et al., 1977; Cohen et al., 1977; Cohen and Siekevitz, 1978; Carlin et al., 1980; Dosemeci et al., 2001; Tao-Cheng et al., 2009; High et al., 2015).

**FIGURE 1:**
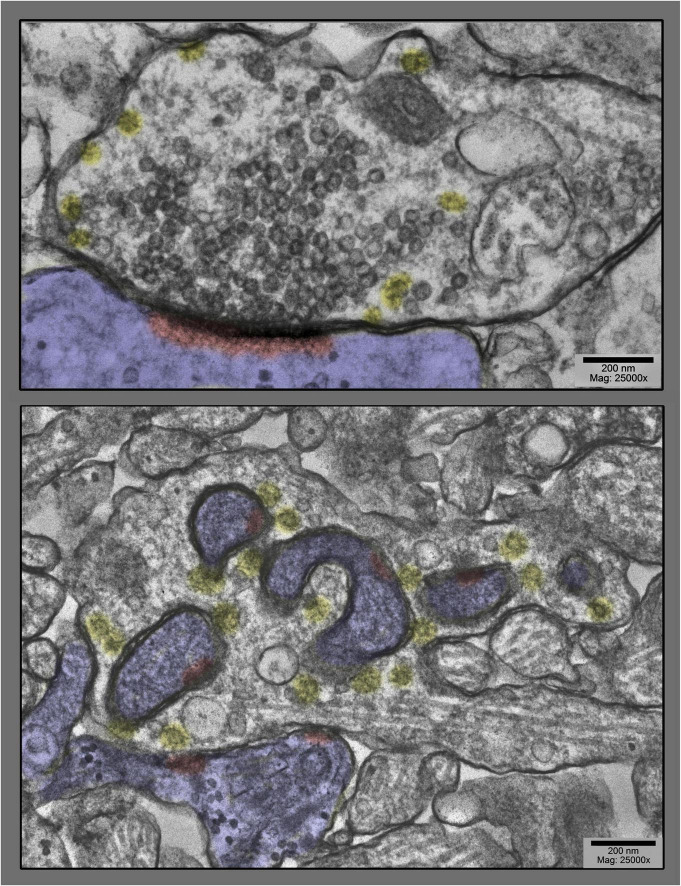
Upper panel shows a prototypical spine synapse from a control hippocampal culture, a culture not stimulated at all. Abundant synaptic vesicles are collected on the presynaptic side, converging on a solid, disk or plaque-like postsynaptic density (highlighted in red, inside the postsynapse, which is highlighted in blue in this and all subsequent figures). Only a few clathrin-coated vesicles are present (highlighted in yellow), and only at the periphery of the vesicle cluster. Lower panel shows, by way of contrast, a dramatically different spine synapse from a hippocampal culture exposed to 0.1 mM La + + + for 20 min at 37°C. Synaptic vesicles are severely depleted, clathrin-coated vesicles (yellow) are unusually abundant, and the postsynapse (highlighted in blue) has “protruded” into the presynapse at several places, dragging along only fragments of the PSD (highlighted in red).

**FIGURE 2:**
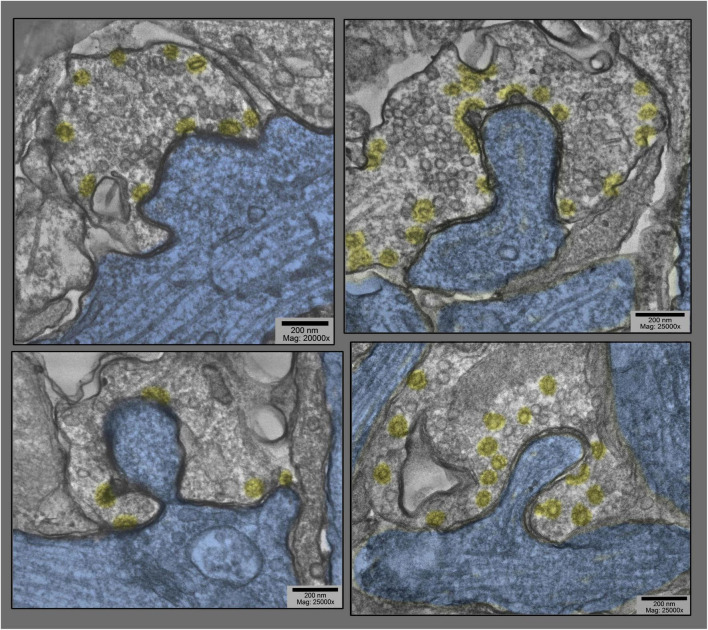
Examples of dendritic shaft synapses in hippocampal cultures exposed to 0.1 mM La + + + for 10–20 min at 37°C. Synaptic vesicles are more or less depleted, but in all cases, clathrin-coated vesicles (presumably involved in the recycling of synaptic vesicle membrane) are increased in abundance, especially along the borders of the postsynaptic “protrusions” (highlighted in blue). Being shaft synapses (thus presumably inhibitory or Gray’s type II), the PSDs are not prominent in these fields and thus are not highlighted in red.

**FIGURE 3:**
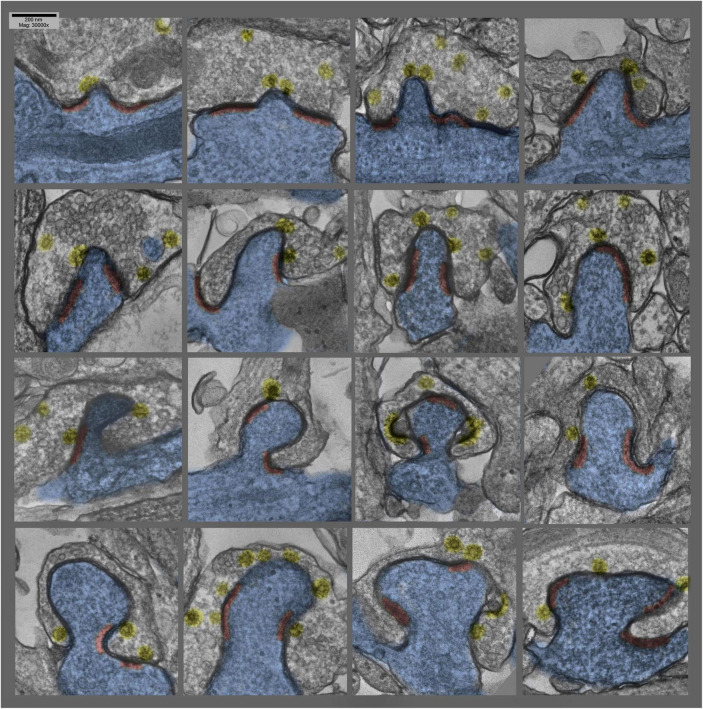
Montage of several different postsynaptic “protrusions” (or presynaptic “hugs,” depending on one’s vantage point) in hippocampal cultures exposed to 0.1 mM La +++ for 10–20 min at 37°C. Synaptic vesicles are more or less depleted, and clathrin-coated vesicles (again highlighted in yellow) are relatively abundant, and PSDs (highlighted in red) are hanging back or are fragmented and “holding on” to the edges of the postsynaptic protrusions.

**FIGURE 4:**
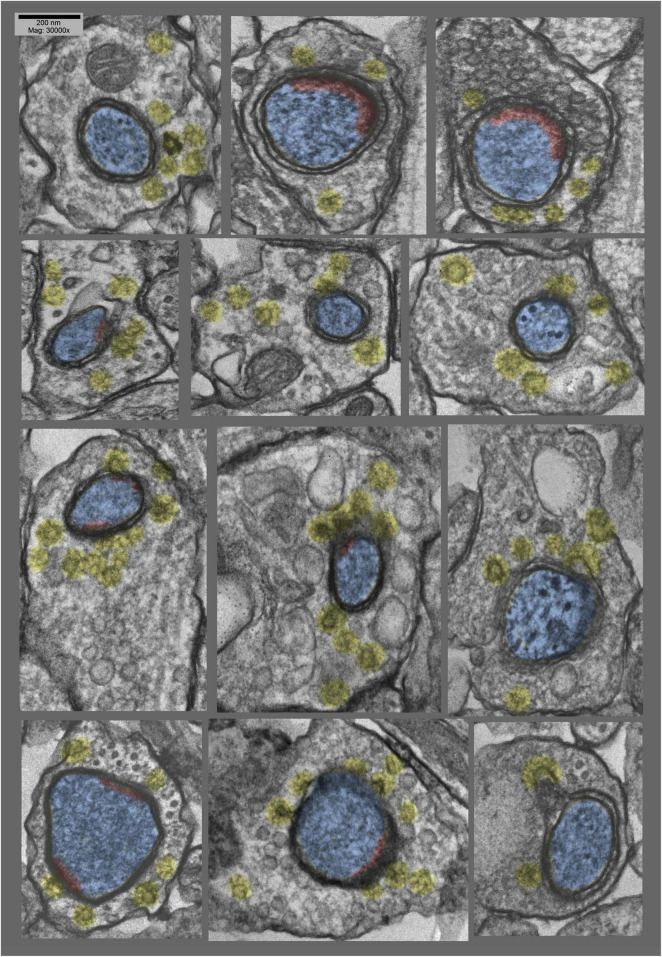
Montage of cross sections of several other postsynaptic “protrusions” or presynaptic “hugs,” from the same hippocampal cultures as in Figure 3 (exposed to 0.1 mM La +++ for 10–20 min at 37°C). Again, it is quite apparent that synaptic vesicles are more or less depleted, compared with the relative abundance of clathrin-coated vesicles (highlighted in yellow). Again, PSDs (highlighted in red) appear to be relatively fragmented or absent from these deeper regions of the postsynaptic involutions.

**FIGURE 5:**
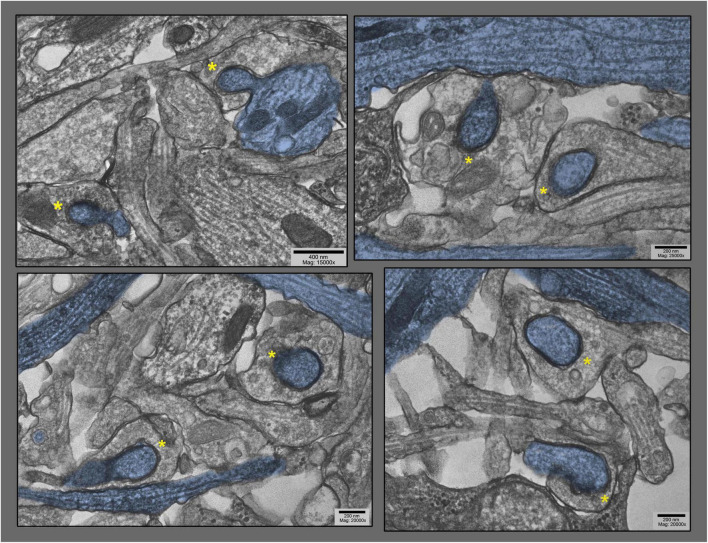
Lower magnification survey views of hippocampal cultures exposed to 0.1 mM La + + + for 10–20 min at 37°C), where only the particular dendrites that are involuting into their apposed synapses are highlighted in blue (other dendrites are also present in these fields). These views were chosen specifically to demonstrate the old adage that George Palade, the great “founder” of biological electron microscopy, always stressed: namely, that if a structural feature could be found *two or more times in one and the same field of view* in the electron microscope, then it must be generally present, and must be not an artifact. Two postsynaptic “protrusions” are indicated by asterisks in each field.

**FIGURE 6:**
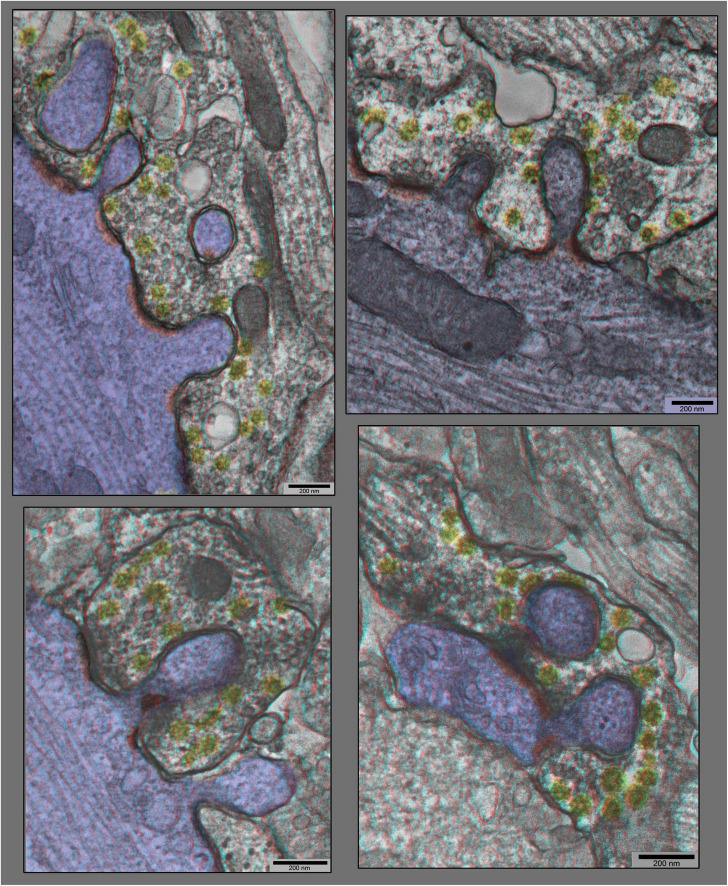
“Anaglyph” 3D views of additional synapses in hippocampal cultures exposed to 0.1 mM La +++ for 10–20 min at 37°C), from plastic blocks that were cut thicker (at 120–150 nm), and photographed at +20° and –20° of tilt in the EM, then superimposed to make the 3–D “anaglyphs”. These require red/green “anaglyph” glasses to fully appreciate, but even without, it is still readily apparent that synaptic vesicles are relatively depleted, coated vesicles are relatively abundant (yellow), and postsynaptic densities are relatively fragmented (PSDs highlighted in red, and dendrites highlighted in blue).

Lanthanum promptly changes the appearance of both bouton-like and plaque-like presynaptic terminals, replacing many of their synaptic vesicles with clathrin-coated vesicles, even in the first 5–10 min after application, and at later times, leaves many of these presynaptic terminals almost empty, or at best, partially filled with irregular membrane forms (as well as residual clathrin-coated vesicles and “empty cages”) (Figures 1–6). Essentially, these are the structural changes that we observed originally at the frog NMJ (Heuser and Miledi, 1971; Heuser, 1976, 1989a,b), which initiated the whole idea that synaptic vesicle membrane could be recycled, and that we have recently been able to observe in mammalian NMJs as well (Heuser and Tenkova, 2020).

### Consequences of Lanthanum’s Stimulatory Effects Projected Onto the Postsynapse

Most unexpected, however, was the dramatic change in the overall configuration of these cultured synapses, a configuration that began to appear in lanthanum at 10 min, and peaked in abundance at 20 min (and seemed to die down by 30 min). This was manifested as a discrete “bulge” of the postsynapse, directly into the midst of the presynaptic terminal (Figures 1–6).

Generally, the postsynaptic protrusions observed in lanthanum occupy a considerable portion of the presynaptic cytoplasm, and draw a considerable mass of postsynaptic cytoplasm into them. In the EM, this bolus of cytoplasm generally appears featureless, or appears finely mesh-like in appearance. It does not look like an active, actin-based growth from the postsynapse, but more like a passive inclusion, only occasionally containing any recognizable postsynaptic membranous organelle.

It is important to consider one clue as to *how or why* these protrusions form in the first place. This comes from closely examining the membrane of the *presynapse* that forms the protrusion (or we could say in the *invagination*, when considered from the presynaptic side). It typically displays all the “spikes” and “clathrin cage fragments” seen in other regions of the presynapse that are undergoing rapid and abundant clathrin-coated vesicle formation. That is, it looks in the EM as if the presynaptic membrane is “committed to endocytosis” in these involutions, or is “trying” to perform endocytosis in the regions that have been drawn inwards into the protrusion.

### Unique Positioning of the Postsynaptic Protrusions and Distortions

Perhaps the most important aspect of these postsynaptic protrusions in the present context, however,—that of considering their possible role in LTP—is that they typically occur *right in the midst* of the PSD. As a consequence, they occasionally drag portions of the PSD inward as they form, depositing these PSD fragments along the “necks” of the invaginations. More often, however, PSD components appear to be excluded from these invaginations—to somehow “hang back”—such that the PSD becomes perforated or partitioned by the invagination.

How or why these invaginations form *right in the midst* of the PSD—rather than around its edges, for example, where one might imagine the membranes to be more “flexible”—is one of the great mysteries that emerges from this study, and remains to be answered.

## Discussion

### Clues About How and Why the Postsynaptic Protrusions Develop

**FIGURE 7:**
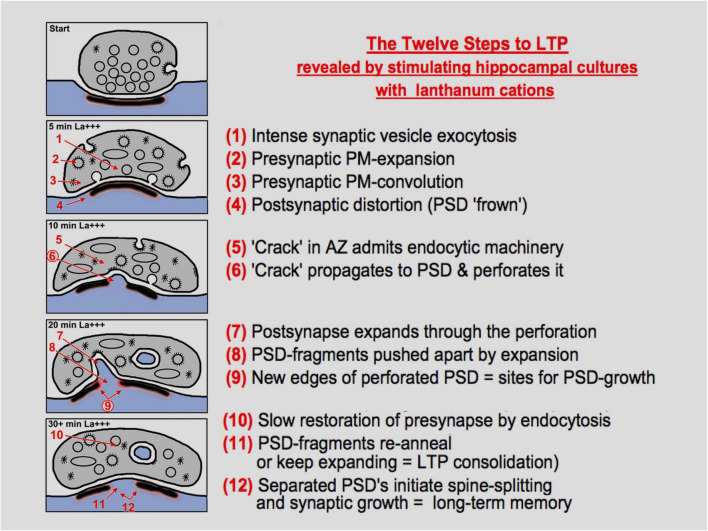
Diagrammatic summary of the observations, interpretations, and hypotheses presented in this study, presented as “twelve steps”, to paraphrase a term from popular culture. The diagram is self-explanatory, but many steps will need to be validated or explained mechanistically by future work, especially the idea that enhanced endocytosis can cause a “break” in the presynaptic active zone (AZ), and that this break can propagate to the PSD to begin the process of perforation or intrusion or spinule formation.

The hypothesis that emerges from these observations is that overly exuberant presynaptic expansion and attempts at endocytosis are “deforming” their active zones and producing the inward membrane bulges that perforate the PSD (Figure 7). Support for this hypothesis came from our attempts to duplicate the effects of lanthanum on these cultures with other sorts of chemical stimulation of them (either by the applying the excitatory neurotransmitter NMDA, or by elevating potassium to depolarize all the cells in the culture). But neither of these forms of stimulation induced any such protrusions or inward invaginations of their postsynapses, but the most they showed was the slight change in PSD curvature that has been reported earlier (Dyson and Jones, 1980; Markus and Petit, 1989; Petit et al., 1989; Markus et al., 1994; Tao-Cheng et al., 2007; Tao-Cheng, 2019). We would argue that this was probably because the synapses in these NMDA or K^+^ -stimulated cultures were not driven out of their “comfort zones,” the zones where they could adequately compensate for their enhanced transmitter release (and their enhanced synaptic vesicle exocytosis) by commensurately accelerating their membrane recycling processes, so that they stayed effectively “in balance,” and did not develop any net accumulation of synaptic vesicle membrane on their surfaces, and thus did not expand in surface area (and consequently, did not attempt to “embrace” the postsynapse or support any postsynaptic protrusions).

Indeed, we may learn someday that the most important reason for why lanthanum stimulation is so effective at expanding the surfaces of nerve terminals and bringing out signs of synaptic vesicle recycling is that it somehow slows down this membrane recycling, at the same time that it stimulates transmitter release, itself. This may someday be explained by several different mechanisms: for example, by some sort of “stiffening” of the presynaptic membrane or by La+++ tending to “glue” the presynaptic membrane to the extracellular matrix, or possibly by blocking sodium and calcium entry through the presynaptic membrane, which may in some way *directly* slow down some aspect of the recycling process. Indeed, there is a large body of evidence, which suggests that clathrin-coated vesicle formation and/or synaptic vesicle recycling is somehow dependent on intracellular Ca^++^ being at just the right level (Henkel and Betz, 1995; Neale et al., 1999; Vogel et al., 1999; Teng and Wilkinson, 2003; Zefirov et al., 2006; Yao et al., 2009; Morton et al., 2015; Miyano et al., 2019; Bourgeois-Jaarsma et al., 2021; Jiang et al., 2021). In any case, it is abundantly clear from the present observations and from the past work that lanthanum somehow creates a greater imbalance between exocytosis and endocytosis than any other form of synaptic stimulation, and consequently, produces the most enhanced accumulation of synaptic vesicle membrane on the presynaptic surface, and thus, the greatest expansion of the presynaptic membrane.

In this respect, these synaptic perturbations in lanthanum-stimulated hippocampal cultures show a remarkable parallel with the abundant, multiple, and florid invaginations of the presynaptic membrane that were seen so many decades ago in frog NMJs treated with lanthanum (Heuser and Miledi, 1971; Heuser, 1976, 1989a,b). These were *also* studded with endocytic profiles, and the thinking at that time was the same as today—that lanthanum caused such an unremitting stimulation of the NMJ that endocytosis became exhausted and “blocked” at that point, or at least greatly slowed down, such that it left much of the discharged synaptic vesicle membrane on the surface of the presynaptic terminal, and thereby *expanding* that surface. A lot of good immunocytochemistry was done in the ensuing decades, which entirely supported this view (von Wedel et al., 1981; Jones et al., 1982; Segal et al., 1985; Robitaille and Tremblay, 1987; Torri-Tarelli et al., 1987, 1990; Valtorta et al., 1988).

The important difference between the changes seen here, in lanthanum-stimulated hippocampal synapses, compared with the old NMJ observations, is that the *postsynapse proper* is pulled into the presynapse. This cannot happen at the NMJ, where the presynapse is separated from the postsynapse by a thick and rigid basal lamina. Instead, at the NMJ, the surrounding Schwann cell gets pulled into the invaginations—or one could say, the Schwann cell ends up “protruding” into the presynaptic terminal. (In this regard, it is worth noting that we observed no such “drawing inward” of surrounding glial processes in any of our lanthanum-stimulated hippocampal cultures. This at least partly due to the simple fact that there are not very many glial processes around the synapses in our cultures, in the first place; and the few glial cells that do happen to be there may have very little “give.”) Quite different is the situation at all NMJs, where Schwann cells totally embrace the whole nerve terminal, except at its immediate contact with the muscle, and where the Schwann cells are highly redundant and “plastic,” so they actively fill in any convolutions of the presynaptic membrane that develop during stimulation. Interestingly, in the primary neuron cultures from the De Camilli lab, their endocytosis-inhibited genotypes show both glial and postsynaptic involutions when the synapses are stimulated (Hayashi et al., 2008; Milosevic et al., 2011, Wu et al., 2014).

### Comparing Classical Postsynaptic “Spinules” With the Protrusions and Distortions Seen Here in Lanthanum

There is no good reason to think that the so-called synaptic “spinules” described in many previous studies of hippocampal synapses (referenced in paragraph 1, above) can or should be differentiated from the *fatter* postsynaptic protrusions into the presynapse that we described here, as being the consequences of lanthanum stimulation. *Neither* type of invagination contains any postsynaptic structure that would suggest they were “active” invasions into the presynapse. In other words, *neither* type contains any signs of actin, nor any other cytoskeletal component might suggest that they actively push their way into the presynapse. Instead, the EMs presented here show clearly that both “spinules” and their fatter counterparts invade regions of the presynapse that show all the signs of being engaged in clathrin-mediated endocytosis. As explained above, this dedication to endocytosis is reason enough to understand why the presynapse should be involuted at those sites. But it also suggests that the spinules and their fatter counterparts, the postsynaptic protrusions, are not *actively splitting* the PSDs as they invade the presynapse. Again, their lack of contractile/propulsive machinery would seem to rule this out. Instead, this PSD splitting appears to be a *passive* process, almost an inadvertent consequence of the presynaptic involution, itself (inadvertent, except that it perhaps was selected by nature to be the fundamental “growth” event of LTP!!).

It is worth stressing here that both “spinules” and the fatter protrusions display uniformly close approximations of pre- and post-membranes, in their midst—the important point being that these are *closer* approximations than those observed at the AZ/PSD differentiations of the synapse *per se*, where so many spanning and attachment molecules are known to be located (and are known to be so abundant and strong that they can even hold the pre- and post-membranes together during homogenization of nervous tissue) (Blomberg et al., 1977; Cohen et al., 1977; Cohen and Siekevitz, 1978; Carlin et al., 1980; Dosemeci et al., 2001). All these specific synaptic attachment proteins have spanning lengths of ∼15–20 nm, which are *greater* than the pre-to-post-membrane separation seen at the protrusions/involutions under question (∼10 nm). Indeed, this 10 nm separation distance that we observed in all the protrusion sites is the normal “minimum” found everywhere in the neuropil of our hippocampal cultures, e.g., between glia and neuronal elements, and between undifferentiated neural elements and each other. (Of course, this “minimum” is not *nearly* close enough to suggest electrical coupling, or anything of that sort.) In any case, this is one more indication that the protrusions are not pulling in the PSDs and their associated attachment proteins with them; rather, they are *splitting* the PSDs, and leaving these attachment proteins behind.

Here, we should add the qualification that despite the obvious structural parallels between synaptic spinules and their fatter counterparts displayed here, nothing in our studies to date would suggest that a precursor/product relationship exists between them. While both seem clearly to be exacerbated or accentuated by synaptic stimulation, the *timing* of this stimulation varies over several orders of magnitude (from just a few minutes in the present study of acute, ongoing chemical stimulation) to hours or longer, in previous studies characterizing the long-term aftereffects of LTP induction, as listed above. In other words, it is not (yet) possible to suggest that spinules grow into fatter protrusions as they invade, or that fat protrusions shrink down to spinules as they withdraw. These possibilities await further experimental discussion and analysis.

## Conclusion and Future Perspectives

The simple observations and interpretations offered here provide a rationale for why such discontinuous or broken-apart PSDs could very likely represent the physical substrate of the temporary “tagging” of synapses that is generally thought to be so important for *initiating* the whole process of LTP (Frey and Morris, 1997, 1998a,b; Martin and Kosik, 2002; Redondo and Morris, 2011; Shires et al., 2012; Evans et al., 2021). This initiation is thought to prepare the synapse for later, long-term enhancement *via* new protein synthesis, which is presumed by all to be the consolidating event that culminates LTP (Bliss and Gardner-Medwin, 1973; Bliss and Lomo, 1973; Carlin and Siekevitz, 1983; Lømo, 2003, 2018; Bailey et al., 2015).

The rationale would be that the breakup of solid plaques or disks of PSD would create *new edges* where “modules” or molecular components of postsynaptic receptors, channels, and signaling molecules could be added, to enlarge or even create completely new PSD plaques. In other words, that the discontinuities and irregularities in the PSD created by the act of perforation, due to the exacerbation of synaptic vesicle recycling in the presynapse, would create additional (and new) *free edges* around the plaques—which originally had, by the simple fact that they were disk-like—had the *minimum* number of free edges possible, for a given collection of receptors (Figure 7).

The logical conclusion to draw from this seems to be that newly generated PSD “free edges” represent the synaptic “tags” that initiate LTP (Frey and Morris, 1997, 1998a,b; Martin and Kosik, 2002; Redondo and Morris, 2011; Shires et al., 2012; Evans et al., 2021). Although we did not attempt in this study to provide the complete structural evidence for this hypothesis, we predict that it should soon become available from many of the new LM and EM methods that are being developed to “tag” various presynaptic and postsynaptic proteins and protein complexes (MacGillavry et al., 2013; Broadhead et al., 2016; Zeng et al., 2016; Biederer et al., 2017; Chen et al., 2018, 2020; Crosby et al., 2019; Trotter et al., 2019; Obashi et al., 2021; Ramsey et al., 2021; Wegner et al., 2022). Here, we focused only on providing direct EM images that demonstrated how (and why) enhanced bursts of presynaptic secretory activity apparently *create* or *cause* the perforation of otherwise plaque-like postsynaptic densities, in the first place.

## Materials and Methods

### Cell Culture

Dissociated cell hippocampal cultures were prepared from papain-dissociated hippocampi, which were harvested from embryonic day 20 rat fetuses, then plated onto confluent glial feeder cultures on 22 mm glass coverslips, and grown for 3–4 weeks before use. Throughout this growth period, the culture medium was half-exchanged 3 × weekly with fresh medium containing MEM (with Earle’s salts, 6 g/L glucose, and 3.7 g/L sodium bicarbonate) supplemented with 5% (v/v) heat-inactivated horse serum, 2% (v/v) fetal bovine serum, and 2 mM Glutamax (all from Life Technologies), along with 136 μM uridine and 54 μM 2-deoxy-5-fluoro-uridine (from Sigma), plus N3 supplement from Sigma (which contains BSA, apotransferrin, putrescine, selenium, T3, insulin, progesterone, and corticosterone) (for further details, see Ransom et al., 1977; Mayer et al., 1989). Throughout this time, the coverslips were maintained in P35 culture dishes in a 36°C incubator with 10% CO_2_.

### Culture Treatments

To conduct the experiments, the culture dishes containing the coverslips were removed from the CO_2_ incubator and *immediately* washed with a bicarbonate and phosphate-free “Ringers” solution. [removing bicarbonate so that the cultures did not alkalinize in room air, with its low CO_2_, and removing phosphate so that the subsequent application of lanthanum did not just precipitate as La(PO_4_)_3_]. Henceforth, they were maintained on a rotating platform in a 37°C water bath. After three more washes in HCO_3_ and PO_4_-free “Ringers,” for a total time of 12 min, the cultures were then exposed to a “Ringers” solution containing 0.1 mM LaCl_3_ (with its usual CaCl_2_ reduced from the usual 2 mM CaCl_2_ to only 1 mM, to minimize any competition of Ca + + with the La + + +. Alternatively, cultures were treated for 5–15 min at 37°C with high K^+^ [a “Ringers” containing 90 mM KCl (whose osmolarity had been compensated by reducing the concentration of NaCl)], or treated for 5–10 min at 37°C with 50–60 μM of N-methyl-D-aspartic acid (NMDA) in normal HCO_3_-free “Ringers,” but containing our usual 2 mM of CaCl_2_ and 3 mM of NaH_2_PO_4_.

### Fixation and Processing

Primary fixation was accomplished by replacing the Ringers solution in the culture dishes with 2% glutaraldehyde, freshly dissolved from a 50% stock (from EMS, Inc.) into a “substitute Ringer’s,” where the normal 5 mM Hepes buffer concentration was increased 6 × for fixation purposes (and NaCl was decreased commensurately, to keep the solution isotonic). Most important at this point was to wash away the La + + + and restore the normal 2 mM calcium in the medium, to prevent any La + + + precipitates in the extracellular spaces of the cultures. (Indeed, this 2 mM calcium is maintained *throughout* primary fixation and postfixation, because we believe that it helps to minimize cellular membrane deterioration).

Immediately after the exchange into the glutaraldehyde, the culture dishes were placed on a vigorously rotating table, and the fixative was exchanged one or two more times, to ensure rapid and uniform fixation. (Even though this aldehyde fixation was probably complete in just a few minutes, we still left the cultures in fixative for another 1–2 h, or even overnight, before initiating postfixation).

The sequence of postfixation was as follows. (This was all done at room temperature, because we believe that cooling biological membranes to 4°C at any time during fixation damages cellular membranes.) First, the glutaraldehyde and Hepes buffer was washed away with 100 mM cacodylate buffer, with two exchanges over a period of at least 15–30 min. (This buffer always contained the same 2 mM Ca + +, in this and all subsequent steps, so it will henceforth be termed “Cacodylate-Ca”). Next, the cultures were postfixed with 0.25% OsO_4_ and 0.25% potassium ferocyanide in Cacodylate-Ca buffer (made fresh, by mixing 0.5% OsO_4_ with 0.5% KFeCN6 immediately before use) for exactly 30 min, no longer. Then, after washing away the OsO_4_ with fresh Cacodylate-Ca buffer (for 5–10 min), the cultures were “mordanted” with 0.5% tannic acid (mw 1,700) in Cacodylate-Ca buffer (making sure to use a batch of tannic acid from EMS or from Polysciences that did not precipitate over time), for 30 min only, no longer. Finally, after washing away the tannate with fresh Cacodylate-Ca buffer, the pH over the cultures was dropped by a brief wash in 100 mM acetate buffer at pH 5.2, to prepare them for “block-staining” with 0.5% uranyl acetate in this acetate buffer (pH 5.2 being the natural pH of dissolved UA, anyway). Then, after this block-staining, they were very briefly washed again in acetate buffer to remove the UA, and finally progressively dehydrated with ethanol in the usual manner (sequential 5–10 min rinses in 50, 75, 95, and 100% ethanol).

### Epoxy Embedding and Thin-Sectioning

Thereafter, the coverslips were removed from the P35 culture dishes to polypropylene bottles, where they could be embedded in Araldite 502 epoxy resin (the old “English Araldite”), *via* an intermediate transfer from ethanol into propylene oxide, then into two-thirds of Araldite and one-third of propylene oxide. (The bottles were needed because the propylene oxide would have dissolved in the original P35 culture dishes). Finally, the fully infiltrated cultures still in their polypropylene bottles were covered with a 10–12 mm deep layer of freshly prepared Araldite 502 epoxy resin and vacuum-embedded in a 70°C vacuum oven, using a strong mechanical pump to draw off all air until the Araldite formed small bubbles (from release of residual propylene oxide and ethanol), and after readmission of air, were left for 24–48 h to fully polymerize.

When fully hardened, the polypropylene bottles were removed from the oven, broken with pliers to release the Araldite blocks, and the blocks were cut with a jeweler’s saw into pieces appropriate for mounting at the desired orientation of the ultramicrotome. Finally, the original glass coverslips on which the cultures were grown were dissolved off of the Araldite by a brief (5–10) min dip into full-strength hydrofluoric acid (47% HF), followed by a number of washes.

Blocks were initially sectioned at 0.5–1.0 μm and stained with 1% toluidine blue and 1% sodium borate in water for 15 s on a hot plate, to examine in the LM and to orient further block-trimming for thin-sectioning.

### Electron Microscopy

Thin sections were cut at 40 nm to obtain the crispest views of membranes, at 90 nm for best general overviews, and at 150–200 nm to obtain 3D information about the overall deployment of synapses in the cultures. Thin sections were picked up on high-transmission fine hexagonal 200-mesh copper grids (made in England by Guilder, Ltd., and sold in the US by Ladd Industries, cat. no. G200HHC), after the grids had been coated with a silver thin film of Formvar, and then carbon-coated by 10 s of vacuum-evaporated carbon, for maximum specimen stability. Finally, sections were stained for 5 min drops of 1% lead citrate (in a closed dish with NaOH pellets around to prevent CO_2_ precipitation of the lead).

They were then examined in a standard TEM operated at 80 kV and mounted with the smallest available objective aperture, for maximum contrast (and maximum removal of chromatic aberration from the thicker sections). They were photographed with the highest resolution digital camera possible, regardless of sensitivity, as such Araldite sections were essentially indestructible and could tolerate endless electron bombardment. (We generally used the AMT “BioSprint” 29 Megapixel Camera, due to its many superior operating features, as well as its very clear 6.5k × 4.5k images). The final digital images were processed and colorized with Photoshop, taking special advantage of its “high-pass” filter when very dark features happened to be located next to very light areas in the images, which made details hard to see. (We typically set the high-pass filter at 40 pixels for our 6,500 × 4,500 pixel AMT images, and layered this filtered image on top of the original image, at 50% density).

### Postscript

We find it absolutely marvelous that over 45 years ago, Sally Tarrant and Aryeh Routtenberg, from Northwestern University’s Neuroscience Laboratory in Chicago, had the prescience and foresight to add the following *tiny and obscure footnote* to their fine study, a study in which they described and discussed “synaptic spinules” in the brains of rats they had prepped for EM by perfusion with Karnovsky’s fixative. The footnote was as follows:

P.S. *“It is also possible that the ‘synaptic spinule’ represents an active synapse and that the presynaptic invagination represents the coalescence of synaptic vesicles and the coated vesicle a device for membrane recycling (Heuser and Reese, 1973). The spine apparatus might contribute to the postsynaptic membrane as it protrudes into the presynaptic membrane invagination.”* Tarrant and Routtenberg (1977).

## Data Availability Statement

The raw data supporting the conclusions of this article will be made available by the authors, without undue reservation.

## Ethics Statement

The animal study was reviewed and approved by the Animal Use and Care Committee of the National Institute of Neurological Disorders and Stroke (NINDS) (Animal protocol Number: ASP1159).

## Author Contributions

JH wrote, read, and approved the final manuscript.

## Conflict of Interest

The author declares that the research was conducted in the absence of any commercial or financial relationships that could be construed as a potential conflict of interest.

## Publisher’s Note

All claims expressed in this article are solely those of the authors and do not necessarily represent those of their affiliated organizations, or those of the publisher, the editors and the reviewers. Any product that may be evaluated in this article, or claim that may be made by its manufacturer, is not guaranteed or endorsed by the publisher.
